# Dual-functional Cu_2_O/g-C_3_N_4_ heterojunctions: a high-performance SERS sensor and photocatalytic self-cleaning system for water pollution detection and remediation

**DOI:** 10.1038/s41378-024-00846-7

**Published:** 2024-12-20

**Authors:** Shuo Yang, Kaiyue Li, Ping Huang, Keyan Liu, Wenhui Li, Yuquan Zhuo, Ziwen Yang, Donglai Han

**Affiliations:** 1 School of Materials Science and Engineering, Changchun University, 130022 Changchun, China; 2 Laboratory of Materials Design and Quantum Simulation College of Science Changchun University, 130022 Changchun, China; 3 School of Materials Science and Engineering, Changchun University of Science and Technology, 130022 Changchun, China

**Keywords:** Nanoparticles, Electronic properties and materials

## Abstract

This study introduces a multifunctional device based on Cu_2_O/g-C_3_N_4_ monitoring and purification p–n heterojunctions (MPHs), seamlessly integrating surface-enhanced Raman scattering (SERS) detection with photocatalytic degradation capabilities. The SERS and photocatalytic performances of the Cu_2_O in various morphologies, g-C_3_N_4_ nanosheets (NSs) and Cu_2_O/g-C_3_N_4_ MPHs with different g-C_3_N_4_ mass ratios were systematically evaluated, with a particular emphasis on the Cu_2_O/g-C_3_N_4_-0.2 MPH, where g-C_3_N_4_ constituted 20% of the total mass. Multiple optical and electrochemical tests revealed that the Cu_2_O/g-C_3_N_4_-0.2 MPH effectively enhances charge separation and reduces charge transfer resistance. The Cu_2_O/g-C_3_N_4_-0.2 SERS sensor exhibited a relative standard deviation (RSD) below 15% and achieved an enhancement factor (EF) of 2.43 × 10^6^ for 4-ATP detection, demonstrating its high sensitivity and consistency. Additionally, it demonstrated a 98.3% degradation efficiency for methyl orange (MO) under visible light within 90 min. Remarkably, even after 216 days, its photocatalytic efficiency remained at 93.7%, and it retained an 84.0% efficiency after four cycles. XRD and SEM analyses before and after cycling, as well as after 216 days, confirmed the structural and morphological stability of the composite, demonstrating its cyclic and long-term stability. The excellent performance of the Cu_2_O/g-C_3_N_4_ MPH is attributed to its Z-type mechanism, as verified by radical trapping experiments. The evaluation of the self-cleaning performance of the Cu_2_O/g-C_3_N_4_-0.2 SERS sensor demonstrated that its Z-scheme structure not only provides excellent self-cleaning capability but also enables the detection of both individual and mixed pollutants, while significantly enhancing the SERS signal response through an effective charge transfer enhancement mechanism.

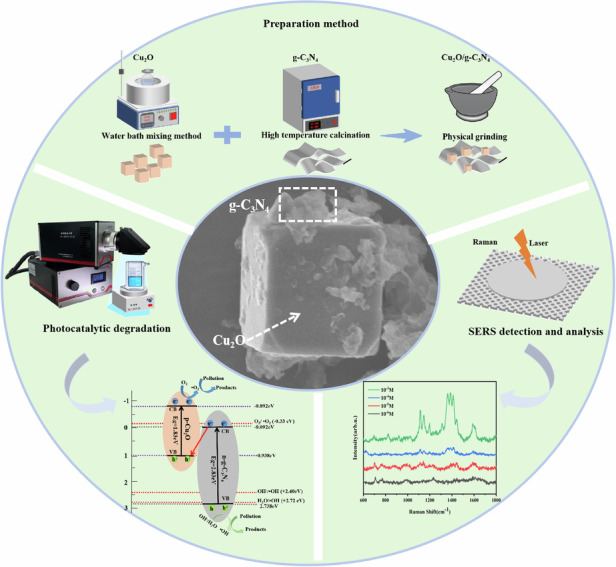

## Introduction

Water pollution has intensified into a significant global environmental issue, driven primarily by the rapid expansion of industrialization and agricultural activities^1^. Large quantities of harmful substances, including dyes, antibiotics, and pesticides, are consistently being released into aquatic ecosystems, either directly or indirectly. Dyes disrupt aquatic habitats and bioaccumulate within the food chain, posing significant toxic risks to human health^2^. Residual antibiotics in water facilitate the development of antibiotic-resistant bacteria, exacerbating antimicrobial resistance and limiting treatment options. Pesticides not only poison aquatic organisms but also leach into groundwater, compromising the safety of drinking water sources^3^. The persistent, concealed and complex nature of these pollutants makes them challenging to completely eliminate or degrade using conventional water treatment technologies. Consequently, the development of multifunctional devices capable of efficient detection and remediation has become essential for effectively addressing water pollution.

Among various water quality monitoring technologies, surface enhanced Raman scattering (SERS) has emerged as a highly effective method for trace pollutant detection due to its high sensitivity and broad-spectrum detection capabilities^4–6^. Traditionally, SERS substrates rely on noble metals such as gold or silver, which enhance Raman signals through surface plasmon resonance^7^. However, the high cost and susceptibility to corrosion of these materials limit their practical application on a large scale. In contrast, semiconductor composite-based SERS substrates exhibit excellent chemical stability and biocompatibility. They are abundant resources, easy to prepare, and cost-effective, significantly reducing detection expenses compared to noble metal substrates, thus improving the accessibility and economic viability of SERS technology. Notably, certain semiconductor SERS substrates, when integrated with photocatalytic technology, can demonstrate substantial photocatalytic degradation potential.

By carefully selecting suitable semiconductor materials, it is possible to achieve dual functionalities within a single material, enabling both pollutant detection and degradation. Among various semiconductor materials, copper oxide (Cu_2_O) stands out as a p-type, narrow-bandgap semiconductor with a broad visible light response spectrum and high solar energy utilization, making it a commonly used photocatalytic material^8^. Additionally, Cu_2_O is both cost-effective and abundant, which further increases its potential for widespread application^9^. In terms of SERS, Cu_2_O not only exhibits intrinsic Raman activity but also possesses surface structures and chemical properties that can significantly enhance the Raman scattering signals of adsorbed molecules. However, Cu_2_O still encounters challenges in practical applications, such as the high recombination of photogenerated electron-hole pairs, which reduces both its photocatalytic and SERS performance, as well as its susceptibility to photocorrosion under light exposure^10,11^. Therefore, enhancing the long-term stability, SERS detection capability and photocatalytic degradation efficiency of Cu_2_O-based multifunctional water quality monitoring and purification devices remains a key focus of current research.

Graphitic carbon nitride (g-C_3_N_4_), an emerging n-type two-dimensional semiconductor, has been identified as an ideal material for modifying Cu_2_O due to its large surface area, excellent chemical stability, low cost, non-toxicity, and suitable bandgap structure^12^. The band alignment between Cu_2_O and g-C_3_N_4_ facilitates efficient electron transfer, reducing electron-hole recombination and thereby enhancing SERS sensitivity and photocatalytic efficiency. Moreover, the incorporation of g-C_3_N_4_ effectively mitigates the photocorrosion of Cu_2_O. Its expanded surface area improves the interaction with target molecules, further amplifying the SERS signals and boosting photocatalytic performance^13^. This composite material system offers new possibilities for developing high-performance multifunctional water quality monitoring devices. Currently, numerous studies have investigated ways to enhance SERS detection or photocatalytic degradation performance through composite approaches. For example, Jianhua Hou et al. ^14^ an oxygen vacancy-rich BiOI/g-C_3_N_4_ composite via a one-pot synthesis at room temperature, achieving a degradation rate under visible light that is 2.6 times higher than that of g-C_3_N_4_. M. Muthukumaran et al. ^15^ developed g-C_3_N_4_@Cu_2_O composites via hydrothermal synthesis, showing superior degradation rates for MB, Rh-B, TB, and Blue-I compared to pure Cu_2_O. Tong Wu et al. ^16^ used Ag nanocrystals onto Au@Cu_2_O for malachite green detection, reaching a limit as low as 10⁻^9^ M. Weiyang Tang et al.^17^ created Au@porous g-C_3_N_4_ substrates, detecting crystal violet at 2.7 × 10⁻^9^ M with an enhancement factor of 6.8 × 10^5^. However, most of the research in the SERS detection field still focuses on noble metal-based composite substrates, with purely inorganic semiconductor composites as SERS sensors remaining relatively uncommon. Furthermore, studies utilizing non-noble metal semiconductor composites for simultaneous SERS detection, photocatalytic degradation, and self-cleaning of SERS substrates are exceedingly scarce.

Inspired by the above ideas, this study developed a multifunctional water quality monitoring and purification p–n heterojunction of Cu_2_O/g-C_3_N_4_ that integrates both SERS detection and photocatalytic degradation functions. The preparation of the Cu_2_O/g-C_3_N_4_ monitoring and purification p–n heterojunctions (MPHs) involved three straightforward steps: First, Cu_2_O microcubes, rounded-edge microcubes, and truncated microcubes were synthesized via a water bath method by adjusting the PVP content, denoted as Cu_2_O MCs, RMCs, and TMCs, respectively. Second, g-C_3_N_4_ with a lamellar flocculent morphology was prepared through high-temperature calcination. Finally, Cu_2_O MCs and g-C_3_N_4_ NSs were physically ground together in varying ratios, where g-C_3_N_4_ accounted for 10% to 50% of the total mass, resulting in composites denoted as Cu_2_O/g-C_3_N_4_-0.1 to 0.5. The SERS and photocatalytic performance of Cu_2_O MCs, g-C_3_N_4_ NSs, and Cu_2_O/g-C_3_N_4_-(0.1–0.5) MPHs were evaluated, with a particular focus on the effect of g-C_3_N_4_ content on the SERS and photocatalytic properties. Special emphasis was placed on assessing the stability, uniformity, self-cleaning ability, and charge transfer mechanism of the Cu_2_O/g-C_3_N_4_-0.2 MPH. In conclusion, the Cu_2_O/g-C_3_N_4_ MPHs exhibit significant potential for applications in photocatalysis, SERS detection, and photocatalytic self-cleaning, providing a promising pathway for the future development of efficient devices for environmental pollution monitoring and purification.

## Results and discussion

### Structural characterization

**Fig. 1:**
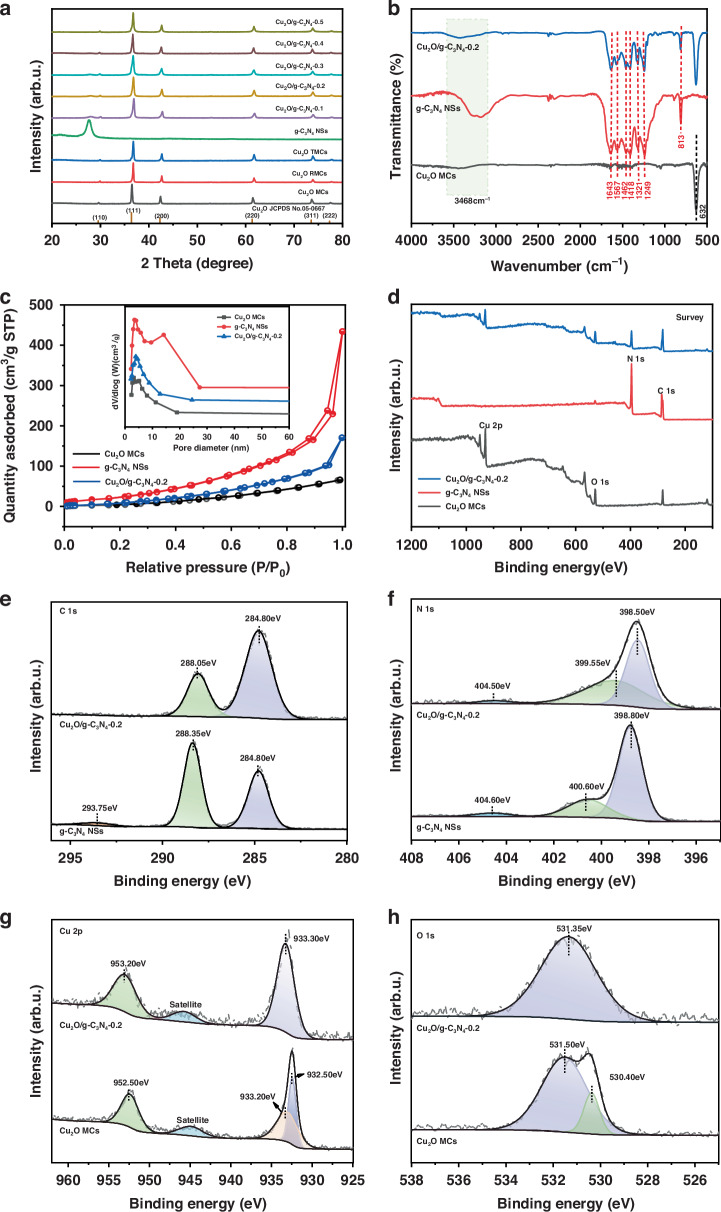
Structural characteristics of prepared samples. **a** XRD patterns of Cu_2_O MCs, Cu_2_O RMCs, Cu_2_O TMCs, g-C_3_N_4_ NSs and Cu_2_O/g-C_3_N_4_-0.1–0.5 MPHs. **b** FT-IR spectra and (**c)** the surface area and pore size distribution of Cu_2_O MCs, g-C_3_N_4_ NSs, and Cu_2_O/g-C_3_N_4_-0.2 MPH. **d** Survey XPS spectra of Cu_2_O MCs, g-C_3_N_4_ NSs, and Cu_2_O/g-C_3_N_4_-0.2 MPH. **e** High-resolution XPS spectra of C 1s, (**f**) N 1s, (**g**) Cu 2p, and **h** O 1 s

As shown in Fig. 1, the structure, chemical composition, surface properties, and chemical states of the synthesized materials were analyzed using X-ray diffraction (XRD), Fourier transform infrared spectroscopy (FT-IR), nitrogen adsorption-desorption isotherms (BET) and X-ray Photoelectron Spectroscopy (XPS). Figure 1a displays the XRD patterns of Cu_2_O MCs, Cu_2_O RMCs, Cu_2_O TMCs and Cu_2_O/g-C_3_N_4_-(0.1–0.5) MPHs. The sharp and narrow diffraction peaks of Cu_2_O MCs, Cu_2_O RMCs and Cu_2_O TMCs appear at 29.5°, 36.4°, 42.3°, 52.5°, 61.3°, 73.5° and 77.2°, corresponding to the (110), (111), (200), (211), (220), (310) and (311) crystal planes, respectively. These peaks match the standard JCPDS card (No. 05-0667) for Cu_2_O, confirming the excellent crystallinity and phase purity of the synthesized Cu_2_O^18^. Furthermore, in Cu_2_O/g-C_3_N_4_-(0.1–0.5) MPHs, a diffraction peak at 27.7°, which is not present in pure Cu_2_O, is associated with the (002) plane of g-C_3_N_4_. This peak is attributed to the interlayer stacking of conjugated aromatic systems^19^, highlighting the successful integration of g-C_3_N_4_ within the heterojunctions.

Figure 1b represents the FT-IR spectra of Cu_2_O MCs, g-C_3_N_4_ NSs and Cu_2_O/g-C_3_N_4_-0.2 MPHs. In the Cu_2_O MCs sample, the peak near 632 cm^−1^ corresponds to the stretching vibration of the Cu–O bond^20^. For g-C_3_N_4_ NSs, characteristic vibration peaks are observed at 813, 1249, 1321, 1418, 1462, 1644, and 3294 cm^−1^, with the 813 cm^−1^ peak representing the characteristic bending vibration of the tri-s-triazine rings units^21^. Peaks between 1100 cm^−1^ and 1700 cm^−1^ are associated with the stretching vibrations of C–N–C bonds^22^. A pronounced peak near 3468 cm^−1^, observed in the spectra of Cu_2_O MCs, g-C_3_N_4_ NSs and the Cu_2_O/g-C_3_N_4_-0.2 MPH, likely results from the stretching vibrations of the O–H bond from adsorbed water^23^. Together with the XRD results, the FT-IR analysis confirms the successful formation of Cu_2_O/g-C_3_N_4_ MPH.

Figure 1c shows the BET isotherms of Cu_2_O MCs, g-C_3_N_4_ NSs and Cu_2_O/g-C_3_N_4_-0.2 MPH. The results indicate that Cu_2_O MCs exhibit a type III isotherm with an H3 hysteresis loop, while g-C_3_N_4_ NSs show a type IV isotherm with an H3 hysteresis loop, indicating slit-like pores formed by layered packing, consistent with the layered structure of carbon nitride. The Cu_2_O/g-C_3_N_4_-0.2 MPH also displays a type IV hysteresis loop. The inset of Fig. 1c shows the pore size distribution for Cu_2_O MCs (9.378 nm), g-C_3_N_4_ NSs (21.352 nm), and Cu_2_O/g-C_3_N_4_-0.2 (10.180 nm), confirming the presence of mesoporous structure, which aligns with SEM observations. Additionally, the specific surface area of the Cu_2_O/g-C_3_N_4_-0.2 MPH (96.469 m^2^/g) lies between that of Cu_2_O MCs (43.352 m^2^/g) and g-C_3_N_4_ NSs (109.249 m^2^/g), indicating that the introduction of g-C_3_N_4_ NSs effectively increases the surface area of the Cu_2_O MCs. This provides more active sites for photocatalytic reactions, enhancing the photocatalytic activity of the Cu_2_O MCs.

Figure 1d presents the XPS survey spectra of Cu_2_O MCs, g-C_3_N_4_ NSs, and Cu_2_O/g-C_3_N_4_-0.2 MPH, while Fig. 1e–h display the high-resolution XPS spectra of C, N, Cu and O elements, respectively. The binding energy values in the XPS spectra were calibrated using the C 1s peak at 284.8 eV as a reference. As shown in Fig. 1e, the peak of g-C_3_N_4_ NSs located at 288.35 eV corresponds to the C–(N)₃ bond, while the peak at 293.75 eV is attributed to π-π* interactions between graphitic layers^24^. In comparison to g-C_3_N_4_ NSs, the peak in the Cu_2_O/g-C_3_N_4_-0.2 MPH, centered at 288.05 eV, shifts by 0.3 eV toward lower binding energy, indicating electron transfer. Figure 1f shows the N 1s spectra of g-C_3_N_4_ NSs and Cu_2_O/g-C_3_N_4_-0.2 MPH. In g-C_3_N_4_ NSs, the characteristic peaks at 398.80 eV, 400.60 eV and 404.60 eV are associated with sp²-hybridized nitrogen C–N = C, tertiary nitrogen atoms (N–(C)_3_) and terminal amino groups (N-C-H), respectively^24^. In Cu_2_O/g-C_3_N_4_-0.2, these binding energies shift by about 0.1–0.3 eV towards lower energy, further confirming electron redistribution. Figure 1g presents the Cu 2p spectra for Cu_2_O MCs and Cu_2_O/g-C_3_N_4_-0.2 MPH. In Cu_2_O MCs, the peak near 933.0 eV is split into two at 932.50 eV and 933.20 eV. The peaks at 933.20 eV and 952.50 eV correspond to Cu 2p_3/2_ and Cu 2p_1/2_, respectively^25^. The peak at 932.50 eV is attributed to Cu^+^(Cu(I)), confirming the presence of Cu_2_O. In the Cu_2_O/g-C_3_N_4_-0.2 heterojunction, the Cu 2p_3/2_ and Cu 2p_1/2_ peaks slightly shift to higher binding energies (933.30 eV and 953.20 eV, respectively), reflecting the electron transfer effect between Cu_2_O and g-C_3_N_4_. The satellite peak near 945 eV indicates the presence of Cu^2+^, suggesting slight surface oxidation of Cu_2_O, leading to the formation of CuO, which can act as a protective layer to enhance the material’s stability and reduce photocorrosion. Figure 1h shows the O 1 s spectra for Cu_2_O MCs and Cu_2_O/g-C_3_N_4_-0.2 MPH. In Cu_2_O MCs, the peak at 530.40 eV is attributed to the Cu-O bond, while the peak at 531.50 eV is related to the free-absorbed OH^−^^26^. In Cu_2_O/g-C_3_N_4_-0.2 MPH, the peak at 531.35 eV shifts by 0.1 eV towards lower binding energy, further confirming electron transfer.

### Morphological characterization

**Fig. 2:**
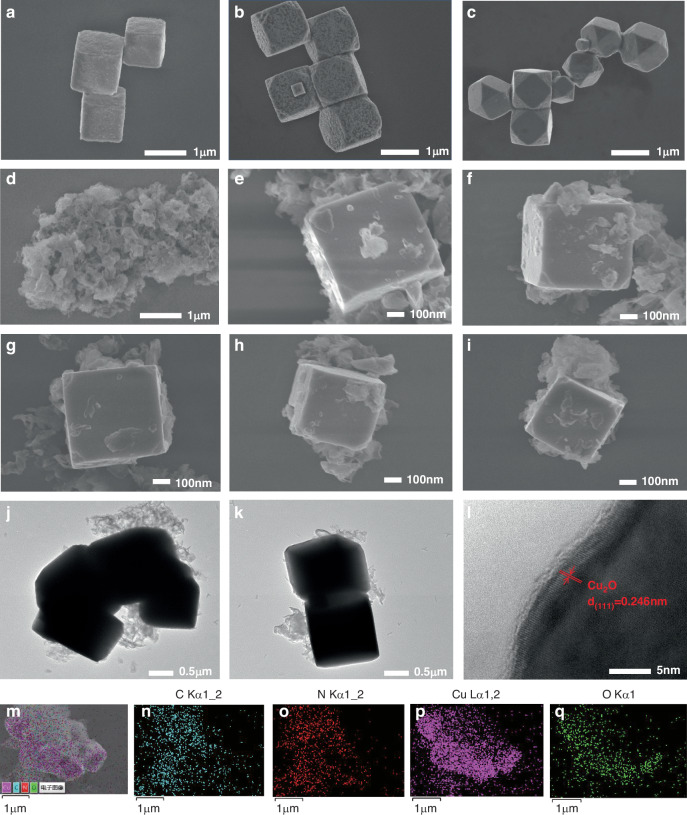
Surface morphology, microstructure and element distribution of prepared samples. **a**–**i** SEM images of Cu_2_O MCs, Cu_2_O RMCs, Cu_2_O TMCs, g-C_3_N_4_ NSs, and Cu_2_O/g-C_3_N_4_-0.1–0.5 MPHs. **j**–**l** TEM and HRTEM images of the Cu_2_O/g-C_3_N_4_-0.2 MPH, **m**–**q** EDS spectra of the Cu_2_O/g-C_3_N_4_-0.2 MPH

Scanning electron microscopy (SEM) was used to observe the morphology of Cu_2_O MCs, Cu_2_O RMCs, Cu_2_O TMCs, g-C_3_N_4_ NSs, and Cu_2_O/g-C_3_N_4_ MPHs, while Transmission Electron Microscopy (TEM), High-Resolution Transmission Electron Microscopy (HRTEM) and Energy Dispersive X-ray Spectroscopy (EDS) were employed to examine the microstructure and elemental composition of Cu_2_O/g-C_3_N_4_-0.2 MPH. As shown in Fig. 2a–c, the addition of 0.55 g of PVP results in minimal truncation of the cubic blocks, allowing the structure to largely retain its cubic geometry, predominantly composed of {100} facets, with particle sizes approximately 1 μm. As the PVP content increases to 0.95 g, truncated cubic blocks with both {100} and {111} facets are observed, and the particle size increases to about 1.2 μm. Further increasing the PVP content to 1.35 g results in a truncated cube structure mainly composed of {111} facets, with particle sizes around 1.5 μm. This morphological change is closely related to the role of PVP, a non-ionic surfactant containing polarizable functional groups such as “−C = O,” which serve as reactive sites^27^. The negatively charged oxygen atoms easily interact with positively charged copper on the surface. As the PVP content increases, the surface energy of the {111} facet decreases, leading to a reduction in {100} facets and an increase in {111} facets. Additionally, PVP acts as a capping agent, promoting charge transfer between g-C_3_N_4_ and Cu_2_O. As observed in Fig. 2d, g-C_3_N_4_ exhibits an irregular sheet-like morphology, with a size of approximately 200 to 500 nm. Figure 2e–i demonstrates that Cu_2_O MCs successfully couples with g-C_3_N_4_ while maintaining their original structure.

The microstructure of the Cu_2_O/g-C_3_N_4_-0.2 MPH was further investigated using TEM and HRTEM. As shown in Fig. 2j, k, the Cu_2_O displays a cubic morphology with a particle size of approximately 1 μm, which is consistent with the SEM observations. The g-C_3_N_4_ exhibits a lamellar flocculent morphology, evenly dispersed on the surface and surrounding the Cu_2_O MCs. These observations confirm the successful formation of the Cu_2_O/g-C_3_N_4_-0.2 MPH. Additionally, the HRTEM analysis of the Cu_2_O/g-C_3_N_4_-0.2 MPH, illustrated in Fig. 2l, reveals lattice fringes with an interplanar spacing of 0.24 nm, which corresponds to the (111) plane of Cu_2_O MCs^28^. However, due to the poor crystallinity or high dispersion of g-C_3_N_4_ NSs, its lattice fringes are not easily discernible^29^. Figure 2m–q shows that the C, N, Cu, and O atoms are uniformly distributed throughout the Cu_2_O/g-C_3_N_4_-0.2 MPH. In summary, the Cu_2_O/g-C_3_N_4_ MPHs were successfully synthesized.

### Photoelectrical performance

**Fig. 3:**
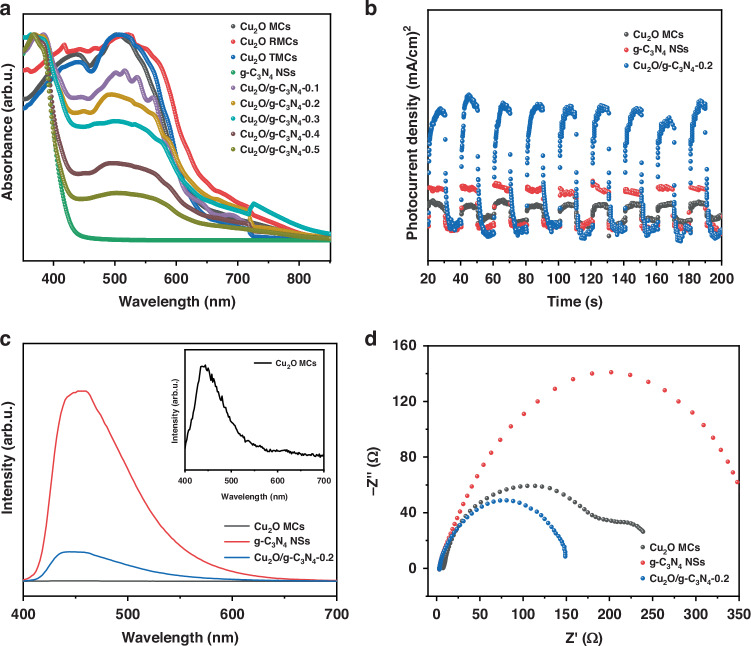
Optical properties and electrochemical performance of prepared samples. **a** UV-Vis diffuse reflectance spectra of Cu_2_O MCs, Cu_2_O RMCs, Cu_2_O TMCs, g-C_3_N_4_ NSs, Cu_2_O/g-C_3_N_4_-0.1–0.5 MPHs. **b** PL spectra, (**c**) *i–t* curves and (**d**) EIS Nyquist plots of Cu_2_O MCs, g-C_3_N_4_ NSs, and Cu_2_O/g-C_3_N_4_-0.2 MPH

Figure 3a shows the UV–Vis diffuse reflectance spectra (DRS) in the presence of Cu_2_O MCs, Cu_2_O RMCs, Cu_2_O TMCs, g-C_3_N_4_ NSs and Cu_2_O/g-C_3_N_4_-(0.1 ~ 0.5) MPHs. The spectra reveal that Cu_2_O MCs, Cu_2_O RMCs and Cu_2_O TMCs exhibit strong absorption peaks in the 400–600 nm range, which aligns with the band structure of Cu_2_O and confirms its activity within the visible light region. The g-C_3_N_4_ NSs displays an absorption edge at 458 nm, corresponding to its bandgap. In the DRS spectra of the Cu_2_O/g-C_3_N_4_-(0.1–0.5) MPHs, as the Cu_2_O content increases, the absorption features of Cu_2_O become more prominent, and the absorption edge of the composites shifts progressively towards longer wavelengths. This red shift indicates that the coupling between Cu_2_O and g-C_3_N_4_ effectively extends the light absorption range of the heterojunctions.

The efficiency of photo-generated charge carrier separation, recombination and transport was investigated using photocurrent intensity (i-t) testing, photoluminescence (PL) spectroscopy, and electrochemical impedance spectroscopy (EIS)^30–32^. As shown in Fig. 3b, the *i–t* of Cu_2_O/g-C_3_N_4_-0.2 MPH is significantly higher than that of Cu_2_O MCs and g-C_3_N_4_ NSs, indicating that the Cu_2_O/g-C_3_N_4_-0.2 MPH has superior photo-generated charge carrier separation efficiency. Figure 3c displays the PL spectra of Cu_2_O MCs, g-C_3_N_4_ NSs, and the Cu_2_O/g-C_3_N_4_-0.2 MPH. As illustrated in the inset of Fig. 3c, the weak emission peak of Cu_2_O MCs observed in the range of 430–570 nm corresponds to bound excitons and defect states localized on their surfaces^33^. In Fig. 3c, the strong peak of g-C_3_N_4_ NSs observed at approximately 450 nm corresponds to its band gap^34^. Compared to g-C_3_N_4_ NSs alone, the markedly reduced PL intensity of the Cu_2_O/g-C_3_N_4_-0.2 MPH indicates that the formation of the heterojunction effectively suppresses the recombination of photo-generated charge carriers, leading to higher charge separation efficiency at the interface and enhancing the photocatalytic performance of the heterojunction. Figure 3d shows the EIS results of Cu_2_O MCs, g-C_3_N_4_ NSs, and the Cu_2_O/g-C_3_N_4_-0.2 MPH, where a smaller Nyquist arc indicates a higher charge transfer rate at the interface. The Nyquist arc radius of the Cu_2_O/g-C_3_N_4_-0.2 MPH is significantly smaller than those of Cu_2_O MCs and g-C_3_N_4_ NSs, suggesting enhanced conductivity and reduced charge transfer resistance. This superior electrochemical performance contributes to the improved photocatalytic efficiency of the heterojunction.

### SERS detection

**Fig. 4:**
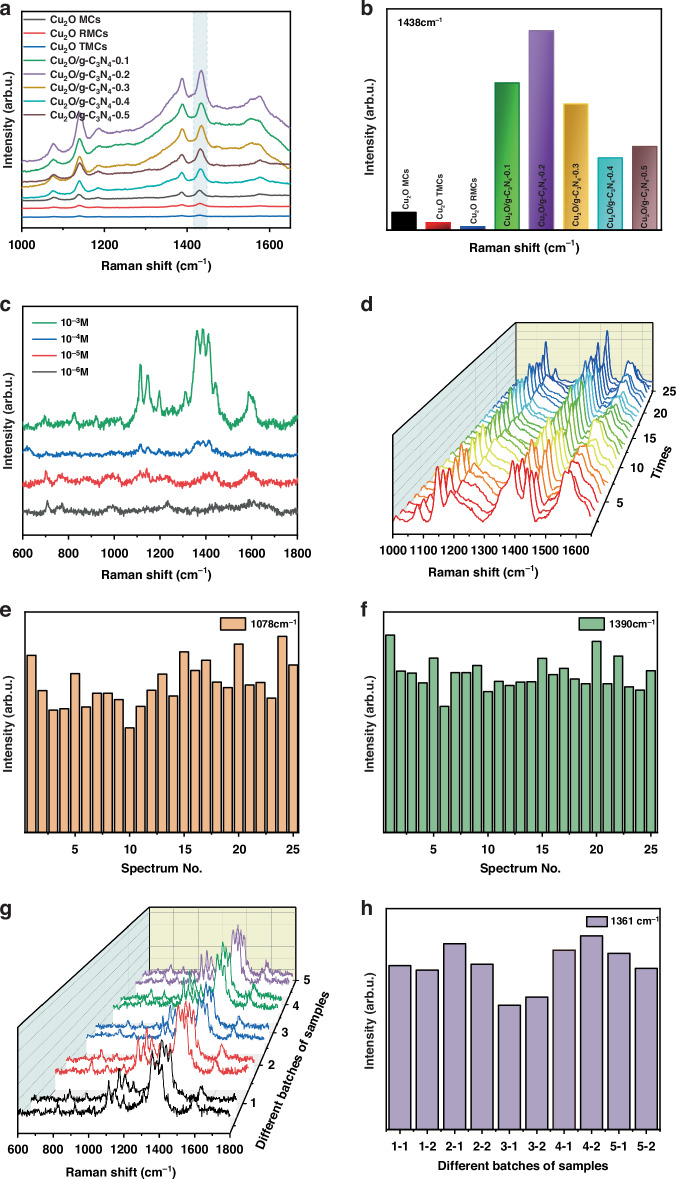
SERS performance of prepared samples. **a** SERS spectra of 4-ATP adsorbed on Cu_2_O MCs, Cu_2_O RMCs, Cu_2_O TMCs, Cu_2_O/g-C_3_N_4_-0.1–0.5 MPHs. **b** SERS signal intensity at 1438 cm^−1^. **c** SERS spectra of Cu_2_O/g-C_3_N_4_-0.2 MPH detecting MO at concentrations of 10^-3 ^M and 10⁻^6^ M. **d** 3D SERS spectra for 25 points on 4-ATP using the Cu_2_O/g-C_3_N_4_-0.2 MPH. **e**, **f** The intensity deviation histograms for SERS intensity at 1078 cm^−1^ and 1390 cm^−1^ across the 25 points, respectively. **g** 3D SERS spectra obtained from separate measurements of MO using five batches of Cu_2_O/g-C_3_N_4_-0.2 MPH prepared at different times. **h** The intensity deviation histograms at 1361 cm^−1^, illustrating the variation in peak intensity across the five batches

Figure 4a presents the preliminary evaluation of the SERS performance of Cu_2_O MCs, Cu_2_O RMCs, Cu_2_O TMCs, Cu_2_O/g-C_3_N_4_-(0.1–0.5) MPHs using 4-ATP as the probe molecule. The results demonstrate that all monomeric Cu_2_O with different morphologies and Cu_2_O/g-C_3_N_4_ MPHs exhibit SERS activity, with the Cu_2_O/g-C_3_N_4_-0.2 MPH displaying the highest SERS signal intensity at 1438 cm^−1^, indicating superior performance (Fig. 4b). The high Raman activity of the Cu_2_O/g-C_3_N_4_-0.2 substrate is primarily attributed to its mesoporous structure and large specific surface area (as shown in Fig. 1c), its enhanced light absorption properties (as shown in Fig. 3a), and its excellent charge separation and transport efficiency (as shown in Fig. 3b–d). The calculated enhancement factor (EF) for the Cu_2_O/g-C_3_N_4_-0.2 substrate is 2.43 × 10^6^. The method for calculating the enhancement factor, along with the relevant parameters and data, can be found in the supporting literature^35,36^.

To explore the practical application potential of the Cu_2_O/g-C_3_N_4_-0.2 substrate as a SERS sensor, it was used for the detection of the dye methyl orange (MO). As shown in Fig. 4c, the peaks at 1120 cm^−1^, 1194 cm^−1^, 1361 cm^−1^, 1389 cm^−1^, and 1418 cm^−1^ are attributed to δ(C-C), υ(=N-Ph_2_), υ(Ph-N), υ(N = N) and υ((C-)SO_2_(-O)), and υ(C-C)^37^, respectively, demonstrating that the Cu_2_O/g-C_3_N_4_-0.2 SERS sensor can successfully detect MO. Furthermore, as the concentration of MO decreases, the SERS intensity of the Cu_2_O/g-C_3_N_4_-0.2 SERS sensor decreases significantly weakens, with a minimum detection limit of 10⁻^6^ M. In addition, substrate uniformity is a critical parameter for ensuring the reliability of a SERS sensor.

To assess the uniformity of the Cu_2_O/g-C_3_N_4_-0.2 SERS sensor, 25 random points on the substrate were selected for SERS measurements. As shown in Fig. 4d, the SERS spectra of 4-ATP from these 25 points reveal no significant variation in peak positions. The relative standard deviations (RSD) of the peaks at 1078 cm^-1^ and 1390 cm^-1^ were calculated to be 14.9% and 9.8% (Fig. 4e-f), respectively, both less than 15%^38^, indicating that the Cu_2_O/g-C_3_N_4_-0.2 SERS sensor possesses excellent uniformity.

To assess the reproducibility and reliability of the Cu_2_O/g-C_3_N_4_-0.2 SERS sensor, five additional experimental batches were prepared and tested for MO detection. Figure 4g, h displays the 3D SERS spectra obtained from two separate measurements of MO using these five batches prepared at different times, as well as the intensity of the peak at 1361 cm⁻¹. As shown in Fig. 4g, the peak positions across all batches are highly consistent, with no significant shifts observed. Figure 4h illustrates that the RSD of the peak intensity at 1361 cm⁻¹ in the MO spectra was calculated to be 7.4%. These findings highlight the excellent reproducibility and reliability of Cu_2_O/g-C_3_N_4_-0.2 MPH as a highly effective SERS sensor for MO detection.

### Photocatalytic performance

**Fig. 5:**
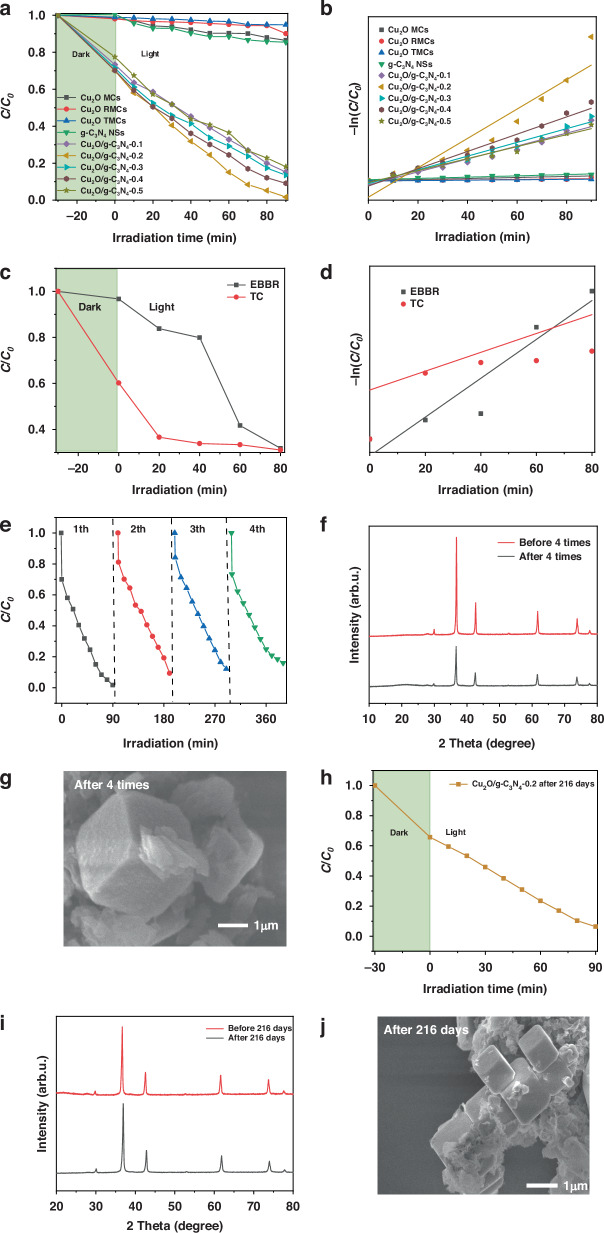
Photocatalytic activity of prepared samples. **a** photocatalytic activity of the Cu_2_O MCs, Cu_2_O RMCs, Cu_2_O TMCs, g-C_3_N_4_ NSs, Cu_2_O/g-C_3_N_4_-0.1–0.5 MPHs for MO degradation under visible light and (**b**) their pseudo-first-order kinetics. **c** The photocatalytic performance of the Cu_2_O/g-C_3_N_4_-0.2 MPH for EBBR and TC degradation and (**d**) their kinetics. **e** The four-cycle MO degradation test using Cu_2_O/g-C_3_N_4_-0.2 MPH. **f** XRD patterns of Cu_2_O/g-C_3_N_4_-0.2 MPH before and after cycling**. g** SEM image of Cu_2_O/g-C_3_N_4_-0.2 MPH after four cycles. **h** The photocatalytic degradation curve of MO by Cu_2_O/g-C_3_N_4_-0.2 MPH after 216 days, and **i-j** XRD and SEM images of Cu_2_O/g-C_3_N_4_-0.2 MPH after photocatalysis at 216 days

The preliminary evaluation of the photocatalytic performance of the Cu_2_O MCs, Cu_2_O RMCs, Cu_2_O TMCs and Cu_2_O/g-C_3_N_4_-(0.1–0.5) MPHs was assessed by degrading MO under visible light. The samples were stirred in the dark for 30 minutes prior to illumination to establish adsorption-desorption equilibrium. As illustrated in Fig. 5a, the photocatalytic degradation efficiencies of Cu_2_O MCs, Cu_2_O RMCs, Cu_2_O TMCs and g-C_3_N_4_ NSs are 14.5%, 10.0%, 5.1%, and 15.11%, respectively, while those of the Cu_2_O/g-C_3_N_4_-(0.1–0.5) MPHs are 79.2%, 98.3%, 81.2%, 87.0%, and 76.6%, respectively. The photocatalytic degradation efficiencies of the Cu_2_O/g-C_3_N_4_ (0.1-0.5) MPHs are significantly higher than those of the individual Cu_2_O samples with three different morphologies and g-C_3_N_4_ NSs, with the Cu_2_O/g-C_3_N_4_-0.2 MPH exhibiting the best performance, achieving a degradation efficiency that is 8 times higher than that of Cu_2_O MCs. This significant improvement is attributed to the synergistic effect between Cu_2_O MCs and g-C_3_N_4_ NSs, which effectively promotes the separation of photo-generated electron-hole pairs, thereby enhancing photocatalytic activity.

Figure 5b demonstrates the quantitative analysis of reaction kinetics using a first-order kinetic model to determine the overall photocatalytic reaction rate. The reaction kinetics are expressed by Eq. (1):

1 $${{{ln}}}({C}_{t}/{C}_{0})=-{Kt}$$

where *K* is the degradation rate constant, *C*_*0*_ is the initial pollutant concentration, and *C*_*t*_ is the pollutant concentration at time t^32^. The first-order reaction rate constants for MO degradation under visible light for Cu_2_O MCs and Cu_2_O/g-C_3_N_4_-0.2 MPH were *K*_Cu2O MCs_ = 0.00135 min^-1^ and *K*_Cu2O/g-C3N4-0.2_ = 0.038 min^-1^, representing a nearly 28-fold enhancement. This significant improvement in the efficiency of the Cu_2_O/g-C_3_N_4_-0.2 MPH can be attributed to the synergistic effect between Cu_2_O MCs and g-C_3_N_4_ NSs. This synergy leads to more efficient separation of photo-generated electron-hole pairs, reduced charge transfer resistance, and improved anti-photocorrosion properties due to the formation of a CuO passivation layer on the material surface. Meanwhile, the Cu_2_O/g-C_3_N_4_-0.2 MPH was further tested for the photocatalytic degradation of eriochrome blue-black R (EBBR) and tetracycline (TC). The results showed that under visible light irradiation, the Cu_2_O/g-C_3_N_4_-0.2 MPH achieved degradation efficiencies of approximately 80% for EBBR and 70% TC within 80 minutes (Fig. 5c), and both followed a first-order kinetic model (Fig. 5d). These findings demonstrate that Cu_2_O/g-C_3_N_4_-0.2 MPH exhibits excellent visible-light photocatalytic degradation performance for multiple pollutants.

Recyclability, structural stability, morphological stability and long-term durability are critical criteria for assessing the quality of a photocatalyst in practical applications. As shown in Fig. 5e, the Cu_2_O/g-C_3_N_4_-0.2 MPH maintained an MO degradation efficiency of 84.0% under visible light even after four cycles. The XRD patterns showed no peak shifts or formation of new phases after cycling, with only a slight decrease in peak intensity indicating a minor reduction in crystallinity, while the morphology remained nearly unchanged (Fig. 5f, g). These results highlight the excellent recyclability, structural stability, and morphological integrity of the Cu_2_O/g-C_3_N_4_-0.2 MPH in photocatalytic applications. Additionally, the photocatalytic performance, structural and morphological stability of the Cu_2_O/g-C_3_N_4_-0.2 MPH were re-evaluated after 216 days. As shown in Fig. 5h–j, the Cu_2_O/g-C_3_N_4_-0.2 MPH maintained a degradation efficiency of 93.7% for degradation MO within 90 minutes under visible light irradiation. The XRD pattern displayed no impurity peaks and the SEM images confirmed that the morphology remained as a Cu_2_O cubic structure combined with lamellar flocculent g-C_3_N_4_. These findings confirm that the Cu_2_O/g-C_3_N_4_-0.2 MPH is stable, reliable, and highly efficient over time.

### Photocatalytic mechanism

**Fig. 6:**
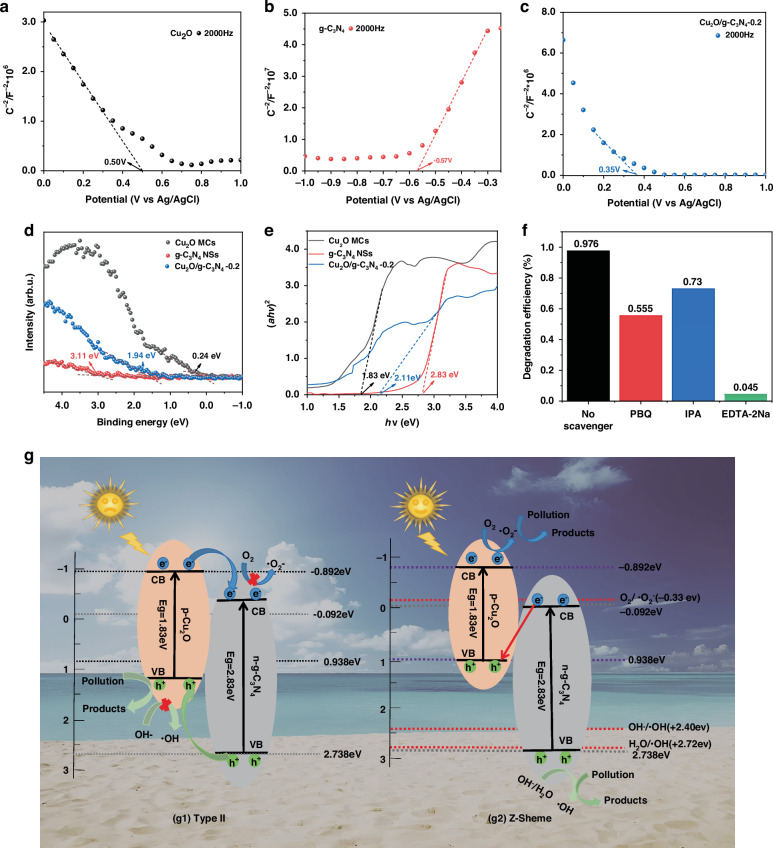
photocatalytic mechanism of the Cu_2_O/g-C_3_N_4_-0.2 MPH. **a–c** MS spectra **d** XPS-VB spectra and **e** Tauc plots of Cu_2_O MCs, g-C_3_N_4_ NSs, and Cu_2_O/g-C_3_N_4_-0.2 MPH. **f** Effects of different radical scavengers on the degradation of MO by the Cu_2_O/g-C_3_N_4_-0.2 MPH. **g** Mechanism for photocatalytic degradation of analytes with Cu_2_O/g-C_3_N_4_-0.2 MPH under visible light

The band structure is crucial for a deeper understanding the interfacial transport of photo-generated charge carriers. The semiconductor types and flat band potentials (*E*_*F*_) of Cu_2_O MCs, g-C_3_N_4_ NSs and Cu_2_O/g-C_3_N_4_-0.2 MPH were analyzed using Mott–Schottky (M–S) curves. As shown in Fig. 6a–c, Cu_2_O MCs show a negative slope and g-C_3_N_4_ NSs show a positive slope, which suggests that Cu_2_O MCs have p-type semiconductor properties and g-C_3_N_4_ NSs have n-type semiconductor properties. Moreover, we can also get the information that the *E*_*F*_ potentials of Cu_2_O MCs, g-C_3_N_4_ NSs, and Cu_2_O/g-C_3_N_4_-0.2 MPH are 0.50 V, −0.48 V, and 0.35 V, respectively. These values were converted to standard hydrogen electrode (NHE) potentials according to the following equation^39^:

2 $${E}_{{NHE}}={E}_{{Ag}/{AgCl}}+0.198\,{\rm{V}}$$

The calculated *E*_*F*_ potentials of Cu_2_O MCs, g-C_3_N_4_ NSs, and Cu_2_O/g-C_3_N_4_-0.2 MPH are therefore 0.698 eV, -0.372 eV, and 0.548 eV vs. NHE (pH = 7.0), respectively. Furthermore, Fig. 6d illustrates that the energy differences between the valence band and *E*_*F*_ for Cu_2_O MCs, g-C_3_N_4_ NSs, and Cu_2_O/g-C_3_N_4_-0.2 MPH were estimated using XPS-VB spectra, with values of 0.938 eV, 2.738 eV, and 2.488 eV, respectively. The bandgap energies (*Eg*) of Cu_2_O MCs and g-C_3_N_4_ NSs were determined using the Kubelka–Munk equation, as shown in the following equation^40^:

3 $${(\alpha h\nu )}^{1/n}=B(h\nu -{E_g})$$

Since both Cu_2_O MCs and g-C_3_N_4_ NSs are direct bandgap semiconductors, n is set to 1/2. As illustrated in Fig. 6e, the Eg of the Cu_2_O MCs and g-C_3_N_4_ NSs are found to be 1.83 eV, 2.11 eV, and 2.83 eV, respectively. According to the following formula^41^:

4 $${E}_{CB}={E}_{VB} - {E_g}$$

The conduction band (*E*_CB_) potentials of the Cu_2_O MCs, g-C_3_N_4_ NSs, and Cu_2_O/g-C_3_N_4_-0.2 MPH were calculated to be -0.892 eV, 0.378 eV, and -0.092 eV, respectively.

To further investigate the effect of different radicals on the photocatalytic degradation of MO, isopropanol (IPA), p-benzoquinone (BQ) and disodium ethylenediaminetetraacetate (EDTA-2Na) were used as scavengers to study the influence of hydroxyl radicals (·OH), superoxide radicals (·O₂⁻), and holes (h^+^) on the Cu_2_O/g-C_3_N_4_-0.2 MPH. Figure 6f shows that the photocatalytic degradation efficiency dropped from 97.6% to 72.97%, 55.48% or 4.5% after the adding of IPA, BQ or EDTA-2Na, respectively. In summary, the photocatalytic degradation of MO by the Cu_2_O/g-C_3_N_4_-0.2 MPH involves three types of active species: h^+^, ·O₂⁻, and ·OH, with h^+^ playing the dominant role, followed by ·O₂⁻ and ·OH.

Based on the previously described band structures and experimental results, two photocatalytic mechanisms under visible light irradiation are proposed in Fig. 6g. As depicted in Fig. 6g1, under visible light excitation, the electrons (e^-^) in the valence bands (VBs) of Cu_2_O and g-C_3_N_4_ are excited to their respective conduction bands (CBs), leaving h^+^ in their VBs. Subsequently, the photo-generated e^-^ migrate from the higher-energy CB of Cu_2_O to the lower-energy CB of g-C_3_N_4_, while holes move from the lower-energy VB of g-C_3_N_4_ to the higher-energy VB of Cu_2_O. This charge transfer process represents a typical type II charge transfer mechanism. Although a type-II mechanism can facilitate efficient charge carrier separation, it is crucial to highlight that the accumulation of trapped electrons and holes may adversely affect the photocatalytic activity in terms of kinetics and thermodynamics^42^. Moreover, the VB of Cu_2_O (0.938 eV) is lower than the oxidation-reduction potentials of OH^−^/·OH (*E* = 2.40 eV vs NHE) and H₂O/·OH (E = 2.72 eV vs NHE)^43^, indicating that Cu_2_O. cannot oxidize OH⁻or H₂O. Furthermore, the CB of g-C_3_N_4_ (−0.092 eV) is more positive than the standard potential for O₂/·O₂− (E = -0.33 eV vs NHE)^44^, meaning it cannot reduce O₂ to generate ·O₂⁻. Consequently, in the type II charge transfer mechanism, the only active species would be h^+^. This contradicts the findings from the radical trapping experiments, suggesting that the type-II mechanism is not applicable in this case. Therefore, the Z-type charge transfer mechanism is proposed, as illustrated in Fig. 6g2. In this mechanism, when the n-type semiconductor g-C_3_N_4_ contacts the p-type semiconductor Cu_2_O, a p–n junction is formed, driving photo-generated e^-^ in the CB of g-C_3_N_4_ to recombine with h^+^ in the VB of Cu_2_O until Fermi level equilibrium is achieved. An internal electric field is subsequently established at the interface, directed from g-C_3_N_4_ to Cu_2_O. This internal field effectively inhibits further recombination of e^-^ in g-C_3_N_4_′s CB with h^+^ in Cu_2_O′s VB, simultaneously leading to the significant accumulation of e^-^ in the CB of Cu_2_O and h^+^ in the VB of g-C_3_N_4_, thereby enhancing their powerful redox capabilities^45^. In this configuration, the photo-generated e^−^ in Cu_2_O (−0.892 eV) possess a sufficiently negative potential to reduce O₂ to ·O₂⁻, while the h^+^ in g-C_3_N_4_ (2.738 eV) have a sufficiently positive potential to oxidize H₂O or OH^−^ to ·OH. Thus, in the Z-type transfer mechanism, the active species are h^+^, ·OH, and ·O₂^−^. This aligns with the radical trapping experiments, confirming that the photocatalytic degradation mechanism follows the Z-type transfer mechanism.

### SERS self-cleaning analysis

**Fig. 7:**
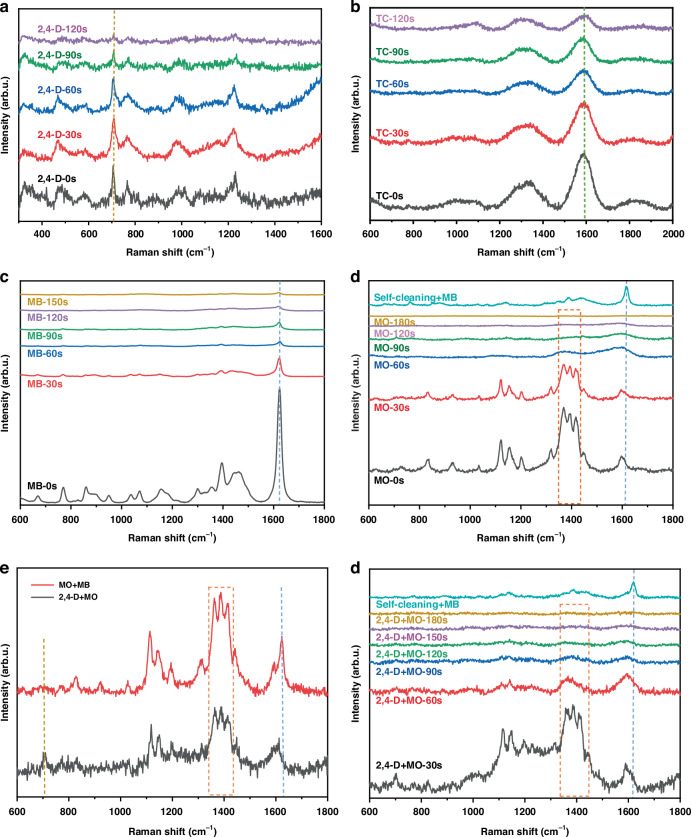
The self-cleaning performance of the Cu_2_O/g-C_3_N_4_-0.2 SERS sensor. **a**–**c** SERS signal variations of 2,4-D, TC and MB (10^-3 ^M) adsorbed on the Cu_2_O/g-C_3_N_4_-0.2 SERS sensor under different illumination times. **d** SERS signal changes of MO on the Cu_2_O/g-C_3_N_4_-0.2 SERS sensor with varying irradiation times, as well as the SERS signals of MB molecules detected on the SERS sensor after the self-cleaning treatment. **e** SERS signals of 2,4-D + MO and MO + MB adsorbed on the Cu_2_O/g-C_3_N_4_-0.2 SERS sensor. **f** SERS signal variations of 2,4-D + MO on the Cu_2_O/g-C_3_N_4_-0.2 SERS sensor under different illumination times, as well as the SERS signals of MB molecules detected on the sensor after the self-cleaning treatment

The self-cleaning performance of the Cu_2_O/g-C_3_N_4_-0.2 SERS sensor was evaluated by monitoring the reduction in intensity of the SERS characteristic peaks of 2,4-D, TC, MB and MO located at 704 cm^−1^, 1585 cm^−1^, 1622 cm^−1^ and 1361 ~ 1418 cm^−1^, respectively, under varying irradiation times (Fig. 7a–f). The results demonstrated that the Cu_2_O/g-C_3_N_4_-0.2 SERS sensor is highly sensitive in detecting individual pollutants such as 2,4-D, TC, MB, and MO. Additionally, it is also capable of accurately detecting and distinguishing mixed pollutants, including 2,4-D + MO and MO + MB mixtures. As shown in Fig. 7a–d and Fig. 7f, as irradiation time increased, the SERS signal intensities of these pollutants progressively diminished, indicating the effective degradation and removal of dye molecules adsorbed on the Cu_2_O/g-C_3_N_4_-0.2 SERS sensor. This observation confirms the superior photocatalytic self-cleaning capability of the sensor. Furthermore, Fig. 7d and Fig. 7f illustrate that after 180 s of irradiation, the cleaned Cu_2_O/g-C_3_N_4_-0.2 SERS sensor was successfully reused for MB detection. The characteristic MB peak at 1622 cm^−1^ reappeared clearly, further demonstrating that the Cu_2_O/g-C_3_N_4_-0.2 SERS sensor was fully regenerated and capable of detecting other pollutants post-cleaning. This further confirms the outstanding reusability and self-cleaning functionality of the Cu_2_O/g-C_3_N_4_-0.2 SERS sensor.

To deepen the understanding of the self-cleaning and reusability mechanisms of the Cu_2_O/g-C_3_N_4_-0.2 SERS sensor, it is crucial to examine the physical and chemical enhancement processes that drive SERS signal amplification^46,47^. In plasmonic nanoparticles, these physical and chemical enhancement mechanisms typically work synergistically. However, for non-plasmonic substrates like Cu_2_O/g-C_3_N_4_-0.2, the SERS effect primarily stems from chemical enhancement^48^. Under visible light irradiation, the strong interaction between Cu_2_O and g-C_3_N_4_ generates numerous active sites on the Cu_2_O/g-C_3_N_4_-0.2 SERS sensor surface, enhancing the adsorption of target molecules. Concurrently, the Z-type heterojunction structure within the Cu_2_O/g-C_3_N_4_-0.2 SERS sensor facilitates efficient separation and transfer of photogenerated e^−^ and h^+^, producing active radicals such as h^+^, ·OH, and ·O₂⁻. These radicals rapidly swiftly target and degrade organic pollutant molecules adsorbed on the surface of the SERS sensor, including MO, 2,4-D, TC, MB and their mixtures. The photocatalytic degradation process, sustained by continuous irradiation, gradually breaks down the dye molecules, leading to the complete removal of the pollutants. This process not only ensures the cleanliness of the sensor surface but also enhances its longevity. Furthermore, the efficient charge carrier separation at the heterojunction interface promotes effective charge transfer, which alters the electronic states and Raman scattering cross-sections of the adsorbed molecules, thereby further amplifying the Raman signals. Consequently, the Z-type heterojunction in the Cu_2_O/g-C_3_N_4_-0.2 SERS sensor not only facilitates self-cleaning through photocatalytic activity but also significantly enhances SERS signals via the charge transfer mechanism.

## Conclusion

This study successfully developed a dual-functional Cu_2_O/g-C_3_N_4_-0.2 heterojunction system that integrates both SERS detection and photocatalytic degradation capabilities, demonstrating its potential as an efficient device for water pollution monitoring and remediation. The Cu_2_O/g-C_3_N_4_-0.2 SERS sensor exhibited high sensitivity with an EF of 2.43 × 10^6^, achieving consistent and reproducible results with an RSD below 15%. Its detection capability extended to various pollutants, including MO, MB, 2,4-D, TC and their mixtures, demonstrating its high sensitivity and broad-spectrum detection potential. The Cu_2_O/g-C_3_N_4_-0.2 MPH also showed superior photocatalytic degradation performance, achieving 98.3% degradation efficiency for MO within 90 minutes under visible light. Remarkably, it retained an 84.0% efficiency even after four cycles and showed a long-term photocatalytic efficiency of 93.7% after 216 days, indicating excellent stability and durability. The study confirmed that the Z-type heterojunction structure plays a critical role in promoting efficient charge separation and preventing recombination, leading to the formation of active species such as h^+^, ·OH, and ·O₂⁻, which drive self-cleaning and photocatalytic degradation. Additionally, the interaction between Cu_2_O and g-C_3_N enhances the adsorption and interaction with target molecules, further amplifying SERS signals. Overall, the Cu_2_O/g-C_3_N_4_-0.2 MPH system not only provides a robust platform for pollutant SERS detection and photocatalytic degradation but also demonstrates exceptional reusability and self-cleaning capabilities. These findings provide a promising foundation for the development of multifunctional, sustainable, and efficient water quality monitoring devices.

## Experimental

### Experimental materials

Copper chloride (AR, CuCl_2_·2H_2_O), sodium citrate (99.0%, C_6_H_6_Na_3_O_7_·2H_2_O), l-ascorbic acid (>99.0%, C_6_H_8_O_6_), polyvinylpyrrolidone ((C_6_H_9_NO)n), and sodium hydroxide (AR, 96%, NaOH) isopropanol (IPA, AR, ≥99.5%), p-benzoquinone (PBQ), ethylenediaminetetraacetic acid disodium salt (EDTA-2Na) were supplied by Aladdin’s Reagent Co. Urea (>99.5%, H_2_NCONH_2_) and anhydrous ethanol (AR, 99%, C_2_H_5_OH) were sourced from McLean’s Reagent Co.

### Preparation of composite materials

#### Preparation of Cu_2_O MCs/RMCs/TMCs

To synthesize Cu_2_O nanoparticles with varying morphologies, different amounts of PVP (0.55 g, 0.95 g, and 1.35 g) along with 0.1 g of C_6_H_6_Na_3_O_7_·2H_2_O were dissolved in 100 mL of a 0.01 M CuCl_2_·2H_2_O solution. Subsequently, 10 mL of 2 M NaOH aqueous solution was added, followed by stirring for 30 min. Following this, 10 mL of 0.6 M l-ascorbic acid was introduced, and the mixture was continuously stirred for an additional 2.5 h. The PVP concentration was found to determine the morphology of the resulting Cu_2_O nanoparticles: 0.55 g of PVP yielded Cu_2_O microcubes (MCs), 0.95 g produced rounded-edge microcubes (RMCs) and 1.35 g resulted in truncated microcubes (TMCs).

### Preparation of g-C_3_N_4_ NSs

10 g of urea was placed in an alumina crucible and heated to 550°C at a rate of 10 °C/min, with the temperature maintained for 2 h in an air atmosphere. The sample was then allowed to cool naturally to room temperature before being washed three times with 0.1 M dilute sulfuric acid, followed by rinsing with deionized water. The washed sample was then dried in a vacuum oven at 80°C for 6 h. Finally, the product was ground using an agate mortar for 15 minutes to obtain pale yellow g-C_3_N_4_ nanosheets (NSs).

### Preparation of Cu_2_O/g-C_3_N_4_ heterojunctions

Cu_2_O/g-C_3_N_4_ heterojunctions were prepared by combining Cu_2_O RMCs and g-C_3_N_4_ NSs in a total mass of 1 part, with g-C_3_N_4_ NSs comprising 0.1, 0.2, 0.3, 0.4, and 0.5 parts of the total mass, respectively. The mixtures were ground in an agate mortar while ethanol was added dropwise during a 90-minute grinding process. The resulting samples were then dried in a vacuum oven at 80°C for 6 h, yielding five distinct Cu_2_O/g-C_3_N_4_ heterojunctions, labeled as Cu_2_O/g-C_3_N_4_-0.1, Cu_2_O/g-C_3_N_4_-0.2, Cu_2_O/g-C_3_N_4_-0.3, Cu_2_O/g-C_3_N_4_-0.4 and Cu_2_O/g-C_3_N_4_-0.5.

### Analytical and characterization methods

The samples’ phase structure was characterized using X-ray powder diffraction (XRD) (Bruker D2, Germany) with Cu-Kα radiation (λ = 1.5406 Å) in the 2θ range of 20°–80°. Functional groups were identified using a Shimadzu IRTracer-00 infrared absorption spectrometer in the 500–4000 cm^-1^ range. The morphology and elemental composition were analyzed using a Hitachi SU8010 scanning electron microscope (SEM) equipped with energy dispersive spectroscopy (EDS). Surface composition and chemical states were investigated through X-ray photoelectron spectroscopy (XPS) with monochromatic Al-Ka radiation (1486.68 eV). The optical properties were measured using UV-visible diffuse reflectance spectroscopy (DRS) with a UV-3600Plus spectrophotometer, spanning the 200–800 nm range. The specific surface area, pore volume, and pore size distribution were measured with a JW-BK200C surface and pore size analyzer (Beijing JWGB Scientific Technology Co., Ltd.). Photoluminescence (PL) spectra were obtained using a Hitachi F-7000 spectrofluorometer with a 320 nm excitation wavelength. Electrochemical measurements were performed on a CHI-760E workstation with a three-electrode system, using an Ag/AgCl reference electrode, a platinum sheet as the counter electrode, and the sample drop-coated onto a 10 mm$\times$10 mm conductive glass substrate. All electrochemical tests were performed in a 1 M Na_2_SO_4_ electrolyte solution. The experimental tests were conducted using a confocal Raman spectrometer manufactured by the French company HORIBA, equipped with a 532 nm laser.

### Photocatalytic degradation experiments

To evaluate the photocatalytic degradation performance of Cu_2_O nanoparticles with different morphologies and Cu_2_O/g-C_3_N_4_ heterojunctions with different mass ratios under visible light, a 20 mg/L methyl orange (MO) solution was prepared. 50 mg of the Cu_2_O/g-C_3_N_4_-0.2 heterojunction was dissolved in 100 mL of deionized water and subjected to ultrasonic agitation for 10 min. The mixture was then stirred at 400 rpm for 30 min to reach adsorption-desorption equilibrium. The beaker was positioned 8 cm from a 300 W xenon lamp equipped with a 420 nm cutoff filter as the visible light source. The suspension was irradiated for 90 minutes, and the change in the absorption peak intensity of MO, EBBR and TC was recorded at 10-minute intervals. Approximately 1 mL of the suspension was sampled and centrifuged at each interval to determine pollutant concentration. The absorbance of the solution was measured using a UV-visible spectrophotometer. Additionally, the Cu_2_O/g-C_3_N_4_-0.2 sample was stored at room temperature in a vacuum drying oven for 216 days. After this period, it was retrieved and subjected to the same photocatalytic degradation experiment for MO under identical conditions to assess the sample’s long-term stability.

### SERS detection

A 10^-3^ M 4-aminothiophenol (4-ATP) solution and a series of MO solutions with concentrations ranging from 10^−^^3^ M to 10^−^^6^ M were prepared. Five milligrams of the test material were immersed in the solutions and incubated overnight at room temperature. Then, 20 µL of the Cu_2_O/g-C_3_N_4_-0.2 heterojunction SERS substrate was gently dropped onto a glass slide and partially dried using a gentle air stream. The glass slide was placed on the detection stage, and the Raman signals of pollutants at different concentrations were measured using a Raman microscope. Raman spectra were collected with a 532 nm excitation wavelength, a 10x objective lens, a laser power of 15 mW, and an exposure time of 10 s.

## Supplementary information

Dual-Functional Cu2O/g-C3N4 heterojunctions: A High-Performance SERS Sensor and Photocatalytic Self-Cleaning System for Water Pollution Detection and Remediation
