# Supplementary material for: Dual-functional Cu2O/g-C3N4 heterojunctions: a high-performance SERS sensor and photocatalytic self-cleaning system for water pollution detection and remediation
Source: Microsyst Nanoeng. 2024 Dec 20;10:198. doi: 10.1038/s41378-024-00846-7 (PMC11659583; doi:10.1038/s41378-024-00846-7)
Supplement: Supplementary file 1 — Dual-Functional Cu2O/g-C3N4 heterojunctions: A High-Performance SERS Sensor and Photocatalytic Self-Cleaning System for Water Pollution Detection and Remediation [file 41378_2024_846_MOESM1_ESM.docx]

**Dual-Functional Cu_2_O/g-C_3_N_4_ heterojunctions: A High-Performance SERS Sensor and Photocatalytic Self-Cleaning System for Water Pollution Detection and Remediation**

Shuo Yang ^a,c^ *, Kaiyue Li ^a,c^, Ping Huang ^a,c^, Keyan Liu ^b^, Wenhui Li ^a,c^ , Yuquan Zhuo ^a,c^, Ziwen Yang ^b^, Donglai Han ^b,^ *

^a^ School of Materials Science and Engineering, Changchun University, Changchun 130022, China

^b^ School of Materials Science and Engineering, Changchun University of Science and Technology, Changchun 130022, China

^c^ Laboratory of Materials Design and Quantum Simulation College of Science Changchun University, Changchun 130022, China

* corresponding author: [yangshuo_2011@163.com](mailto:yangshuo_2011@163.com) (Shuo Yang); dlhan_1015@cust.edu.cn (Donglai Han);

Tel.: +86 0431-85250410; fax: +86 0431-85250410.

Contributing authors: [lkaiyue29@163.com](mailto:lkaiyue29@163.com) (Kaiyue Li), huangping_2023@163.com (Ping Huang), liukeyan0910@163.com (Keyan Liu), [19810911969@163.com](mailto:19810911969@163.com) (Wenhui Li), [zyq19942867075@163.com](mailto:zyq19942867075@163.com) (Yuquan Zhuo), yzw123456789yzw@163.com (Ziwen Yang).

Table 1-1 SERS Band Information of 4-ATP:

| Wavenumber (cm⁻¹) | Spectral band information |
| --- | --- |
| 1078 | υCS(a1) |
| 1141 | δCH(b2) |
| 1177 | δCH(a1) |
| 1390 | υCC+δCH(b2) |
| 1438 | υCC+δCH(a1) |
| 1572 | υCC(b2) |

To more accurately evaluate the performance of the Cu_2_O/g-C_3_N_4_-0.2 composite material as a SERS substrate, the SERS enhancement factor (EF) was calculated using equation (1-1).

$EF=\frac{I_{SERS}/N_{SERS}}{I_{Raman}/N_{Raman}} =\frac{I_{SERS}}{I_{Raman}} \times\frac{S_{laser}\times h\times\rho\times A\times N_{A}}{f\times M\times S_{laser}}$ (1-1)

Where $I_{SERS}$ and $I_{SERS}$ represent the SERS intensity and normal Raman signal intensity of 4-ATP adsorbed on the Cu_2_O/g-C_3_N_4_-0.2 MPH, respectively. $N_{SERS}$ and $N_{Raman}$represent the concentrations of 4-ATP for the SERS and normal Raman scattering, respectively. $S_{laser}$​ is the laser spot area during the Raman scan, *h* is the effective layer depth, N_A_ is Avogadro's constant, ρ is the density of 4-ATP, A is the surface area of a single 4-ATP molecule, f is a constant, and M is the relative molecular mass of 4-ATP.
